# Coherent response of the electronic system driven by non-interfering laser pulses

**DOI:** 10.1038/s41467-022-30768-9

**Published:** 2022-06-09

**Authors:** Tobias Eul, Eva Prinz, Michael Hartelt, Benjamin Frisch, Martin Aeschlimann, Benjamin Stadtmüller

**Affiliations:** 1 Department of Physics and Research Center OPTIMAS, University of Kaiserslautern, Erwin-Schrödinger-Straße 46, 67663 Kaiserslautern, Germany; 2 Institute of Physics, Johannes Gutenberg University Mainz, Staudingerweg 7, 55128 Mainz, Germany

**Keywords:** Nonlinear phenomena, Ultrafast photonics, Electronic properties and materials

## Abstract

The strength of light–matter interaction in condensed matter is fundamentally linked to the orientation and oscillation strength of the materials’ optical transition dipoles. Structurally anisotropic materials, e.g., elongated molecules, exhibit optical transition dipoles with fixed orientations that govern the angular-dependent light–matter interaction. Contrary, free electron-like metals should exhibit isotropic light–matter interaction with the light fields dictating the orientation of the optical transition dipoles. Here, we demonstrate that an anisotropic direction of the optical transition dipoles even exists in highly free electron-like noble metal surfaces. Our time- and phase-resolved photoemission experiment reveals coherent interference effects on the (110)-oriented silver surface after optical excitation with two non-interfering cross-polarized pulses. We explain this coherent material response within the density matrix formalism by an intrinsic coupling of the non-interfering light fields mediated by optical transition dipoles with fixed orientations in silver.

## Abstract (web summary)

Light–matter interaction is expected to be isotropic in free-electron-like materials. Here, by using time- and phase-resolved photoemission, the authors observe signatures of an anisotropic interaction on a noble metal surface, that can only be accounted for by optical transition dipoles with a fixed orientation.

## Introduction

The interaction of light with matter is the starting point of countless fundamental, and application relevant phenomena in condensed matter systems. It determines the speed and efficiency of coherent control protocols designed to manipulate the charge and spin order of magnetic, correlated, or other quantum materials and plays an essential role for the efficient light-to-charge carrier conversion in light harvesting processes^1–6^. For instance, it is responsible for quantum coherence phenomena that emerged as one of the key properties for efficient charge separation, charge transfer, and the generation of long-lived excitons in matter^7–13^.

On a rather fundamental level, the efficiency of the light–matter interaction in all matter is governed by the optical transition dipoles (OTDs), which are characterized by their oscillator strength as well as their orientation within the material. Both properties of the OTDs depend on the shape and symmetry of the electronic wave functions that are involved in the light absorption process and the corresponding optical transition. This can lead to significant differences in the orientation of the OTDs depending on the structural properties of the materials.

In strongly anisotropic systems such as planar aromatic molecules or 2D van der Waals layers, the orientation of the OTD closely relates to the high symmetry directions or anisotropy axis of the materials (e.g., the molecular orbitals of a photosynthetic protein or organic molecules after deposition on an electrode material) and can be identified for example with ultrafast X-ray scattering^14^ or spectroscopy techniques^15–18^.

For simple metals, however, the orientation of the OTDs is usually neglected when describing the efficiency of light–matter interaction. This approximation emanates from the fact that simple metals are nature’s closest realization of the nearly free electron gas. In this model, the electronic properties are isotropic, which reflects in several characteristics of metals, such as the plasma frequency and the plasmon dispersion, which are independent of lattice and surface orientations. In particular, band structure effects typically present only small perturbations to the free electron gas model^19^.

In contrast to the commonly established state of understanding, we demonstrate in our work the importance of the orientation of OTDs for the light–matter interaction in simple metals. We find severe deviations from the isotropic nature of the free electron gas even for a simple noble metal surface such as Ag(110), which manifests itself as a coherent response of the electronic system by the absorption of two orthogonal light pulses. To this end, we employ non-linear, two-photon absorption and monitor the material response with the resulting photoemission process. The technique serves as an ideal probe for light–matter interactions in the range of visible light, which lies at the core of most optical manipulation applications. Our experimental findings cohere with the theoretical description of the two-photon photoemission process in the density matrix formalism, which, in concurrence with the optical Bloch equations, successfully disentangled electronic quasi-particle lifetimes in the past^20–23^. Here, however, it will allow us to uncover the role of the orientation of the OTDs for the light absorption in a simple metal.

## Results

**Fig. 1:**
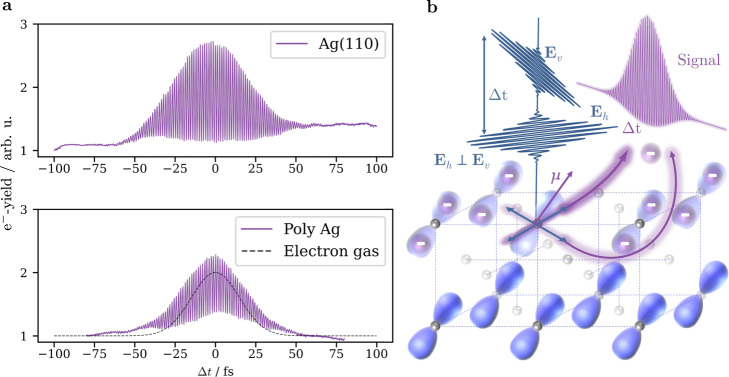
Cross-polarized two-photon photoemission traces of a Ag(110) and a polycystalline silver surface. **a** Energy- and momentum-integrated cross-correlation traces on Ag(110) (top) and polycrystalline silver (bottom) showing interference patterns despite the non-interfering pump-probe excitation scheme. The black dashed curve represents the expected behavior in the case of a free electron gas. **b** Schematic of the coupling between the perpendicular electric fields and the optical transition dipole $\overrightarrow{\mu }$, which leads to coherent photoexcitation for the field components overlapping with the dipole.

The starting point of our discussion is the coherent response of the electron system of different surfaces of the simple metal silver after optical excitation with cross-polarized fs light pulses in the visible range (*E*_*hν*_ = 3.1 eV). Figure 1a shows the time-dependent photoemission traces of our time-resolved interferometric photoemission experiment for Ag(110) (top) and a polycrystalline Ag surface (bottom). For both surfaces, we detect a coherent interference pattern in the photoemission signal during the time steps of the temporal overlap of both cross-polarized pulses, despite their inability to interfere with each other in free space.

This observation is very surprising as it clearly contradicts the isotropic nature of the free electron gas. In that very simple model, the exciting light pulses determine the electronic response of the free electron gas system (e.g., by introducing oscillations along the polarization direction) and dictate the orientation of the OTDs by the direction of their polarization. Accordingly, the OTDs of two cross-polarized pulses would be orthogonal to each other. This would prevent a coherent response of the electronic system of an ideal free electron gas material to the optical excitation with two cross-polarized light pulses, resulting in the simulated black dashed curve in the bottom of Fig. 1a.

In this regard, our experimental findings clearly point to an anisotropy in the electronic response and to a fixed orientation of the OTDs in a simple noble metal. Figure 1b depicts a scenario for an excitation with two cross-polarized pulses *E*_*h*_ (horizontal in-plane polarization) and *E*_*v*_ (vertical in-plane polarization). In this model, the orientation of the OTDs are determined by the bonding orbitals that mediate the chemical interaction between neighboring atoms located on the fcc lattice of Ag. In this case, both pulses *E*_*h*_ and *E*_*v*_ can couple to the same OTD as long as the projection of their electric field vector onto the direction of the OTD is non-zero. The photoelectrons subsequently excited by the individual pulses are therefore still able to coherently interfere. By scanning the time delay between the pulses, we record non-linear correlation curves, where the alignment of the orbitals along the surface is responsible for the existence or absence of a coherence pattern as well as the relative height of the correlation trace.

However, as we will outline in the following, this interference pattern results from two side-by-side mechanisms, namely the coupling of the two pulses to the OTDs (i) and additionally an ultrafast switching of the effective polarization of the superimposed and phase-stabilized pulses (ii). In the present work, we discuss how to identify and disentangle both mechanisms as well as how to extract the orientation of the OTDs from these cross-polarized interferometric experiments. We exemplify why only the second mechanism is present in the case of polycrystalline silver in Fig. 1a (bottom) and illustrate how phase-averaging of the interferometric measurement yields the orientation of the OTDs in anisotropic systems like Ag(110) (top), Au(100) and Cu(110). Supplementary Figures 2–3 show additional data for the latter two systems. Our findings emerge from the theoretical description of the cross-polarized two-photon photoemission process in the density matrix formalism, which we present in the following sections.

### Two-photon photoemission in the density matrix formalism

**Fig. 2:**
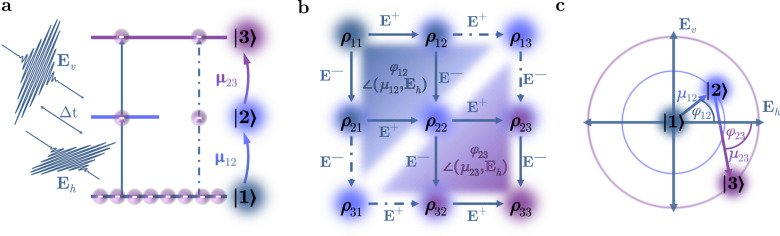
Cross-polarized two-photon photoemission in the density matrix formalism. **a** Two-photon photoemission in a three-level system via real or virtual intermediate states for non-vanishing dot product $\overrightarrow{E}\cdot \overrightarrow{\mu }$. **b** Illustration of the two-photon photoemission process in the density matrix for real (*ρ*_22_, inner pathways) and virtual (*ρ*_13_, *ρ*_31_, outer pathways) intermediate states. Electrons are excited into the final state along every possible pathway of the matrix under all permutations of the electric fields. **c** Direction of the in-plane electric fields with regard to a fixed orientation of the optical transition dipoles yielding the angles *φ*_12_ and *φ*_23_, which have to be applied accordingly along the different pathways.

We describe the non-linear photoemission process starting with a general quadratic correlation trace (see Eq. (1)) and translate that into a density matrix for a three-level system (see Fig. 2). This approach has already been outlined for the case of indistinguishable pulses^24,25^, as well as the mixture of a plasmon field and a light field^25^. In this work, we first have to reintroduce this general description of the two-photon photoemission process in order to comprehensively disclose our adaptation of this theory for the case of two cross-polarized pulses. The two-photon photoemission signal *S* is expressed by the time integral

1 $$S({{\Delta }}t) 	=\int\nolimits_{-\infty }^{+\infty }{\left[{E}_{a}(t)+{E}_{b}(t-{{\Delta }}t)\right]}^{4}\,{{{{{\rm{d}}}}}}t,\\ {E}_{a}(t) 	={A}_{a}(t)\cdot \cos (\omega t),\\ {E}_{b}(t) 	={A}_{b}(t-{{\Delta }}t)\cos (\omega (t-{{\Delta }}t)),$$

that depends on the time delay Δ*t* between the two universal pulses *E*_*a*_, *E*_*b*_. The two pulses consist of the time-dependent amplitudes *A*(*t*) and the laser frequency *ω*. It is worth noting that we explicitly omit the material response function that mainly describes the energy- and momentum-dependent quasi-particle lifetime of excited electrons in this system. Finite lifetimes of the hot electrons cause an increase of the full width at half maximum (FWHM) of correlation traces^24,26^, which is independent of the coherent interference effects that are crucial in this work. The excitation from the initial $\left|1\right\rangle$ to the final state $\left|3\right\rangle$ via a real or virtual (strongly detuned) intermediate state $\left|2\right\rangle$ is governed by the OTD elements ${\overrightarrow{\mu }}_{12}$ and ${\overrightarrow{\mu }}_{23}$ as depicted in Fig. 2a). The OTDs consist of an amplitude dependent on the polarizability of the material as well as an orientation, which depends on anisotropies (e.g., structural, magnetic etc.) in the material. In our work, we solely focus on effects induced by the anisotropies and do not explicitly consider the amplitude of the OTD moments.

The excitation process can be visualized as a combination of steps between the population states (diagonals) and the coherences in the mixed states (off-diagonals) in the density matrix. This is depicted in Fig. 2b), where each step is annotated with an electric field *E* with a positive or negative phase depending on the step direction. However, both electric fields *E*_*a*_, *E*_*b*_ are simultaneously present on the sample during the acquisition time of the experiment. Hence, the photoelectron could be excited either from the two pulses individually or their superpositions and each step has to be evaluated for all possible permutations of *E* → *E*_*a*_ or *E* → *E*_*b*_. In total, this amounts to 96 possible excitation pathways, 64 populating a real intermediate state *ρ*_22_ and 32 exciting a coherent polarization *ρ*_31_, *ρ*_13_, which we denote as the virtual intermediate state. The phase results from the electric field expressed with its Euler’s formula

2 $$E(t)=\frac{A(t)}{2}{{{{{\rm{e}}}}}}^{+{{{{{\rm{i}}}}}}\omega t}+\frac{A(t)}{2}{{{{{\rm{e}}}}}}^{-{{{{{\rm{i}}}}}}\omega t}={E}^{+}+{E}^{-},$$

so that an electron is excited above the vacuum energy by the combination of four fields *E*^*p*^ with all possible permutations of the fields *E*_*a*_ and *E*_*b*_ as well as the phases *p* ∈ {+, −}. By taking a closer look at Fig. 2b it becomes evident, that only the pathways involving two times the positive and two times the negative phase in any order lead to the final state.

Mathematically, the integral

3 $${S}_{k,l,m,n}({{\Delta }}t) 	=\int\nolimits_{-\infty }^{+\infty }{E}_{k}^{\pm }({\tau }_{k}){E}_{l}^{\pm }({\tau }_{l}){E}_{m}^{\pm }({\tau }_{m}){E}_{n}^{\pm }({\tau }_{n})\,{{{{{\rm{d}}}}}}t.\\ {\tau }_{k} 	=\left\{\begin{array}{ll}t \hfill&k\,\widehat{=}\,a\\ t-{{\Delta }}t &k\,\widehat{=}\,b\end{array}\right.$$

with the permutation indices *k*, *l*, *m*, *n* ∈ {*a*, *b*}, defines an individual pathway and the sum over all individual pathway integrals constitutes the total correlation trace (1). On closer examination, the individual pathways exhibit constructively or destructively interfering phase terms of the electric fields depending on the phase combinations ± of the four electric fields in (3). This interference modulates the pathway contribution to the total signal with a multiplicative factor e^*n*⋅i*ω*Δ*t*^ oscillating with multiples of the laser frequency (*n* ∈ {0, 1, 2}). Therefore, we order the contributions to the total two-photon photoemission signal by their oscillating component *nω* into the so-called *oscillating terms*, which are responsible for the coherent interference pattern we see in an interferometric measurement. These terms are usually referred to as the 0*ω*, 1*ω*, 2*ω* components and the following section outlines their interplay with the OTDs in a cross-polarized excitation scheme marking the first mechanism (i) mentioned above.

### Optical two photon transitions with cross-polarized light pulses

In the next step, we specifically introduce the orientation of the OTDs into the density matrix formalism and evaluate the corresponding equations for two cross-polarized light pulses, as suggested in ref. ^24^. Figure 2c shows the excitation via the transition dipoles in relation to the in-plane orientation of the exciting fields. Here, we assume an arbitrary but fixed directivity of the OTDs by introducing the angles *φ*_12_ and *φ*_23_ between the electric field of one of the pulses ${\overrightarrow{E}}_{h}$ and the OTD of the respective transition into the intermediate or the final state. Since these transitions occur individually, these two angles are in general independent of each other. When calculating the individual pathways in Eq. (3), these angles have to be included for each step by redefining the electric fields to

4 $${E}_{a}\mapsto {E}_{h}^{\pm } 	=\frac{1}{2}{A}_{h}(t)\cdot {{{{{\rm{e}}}}}}^{{\pm} {{{{{\rm{i}}}}}}\omega t}\cdot \cos ({\varphi }_{ij})\\ {E}_{b}\mapsto {E}_{v}^{\pm } 	=\frac{1}{2}{A}_{v}(t-{{\Delta }}t)\cdot {{{{{\rm{e}}}}}}^{\pm {{{{{\rm{i}}}}}}\omega \left(t-{{\Delta }}t\right)}\cdot \sin ({\varphi }_{ij}),$$

with the angle depending on the transition indicated in Fig. 2b, while the respective trigonometric function addresses the perpendicularity of the two pulses. We renamed the amplitudes *A*_*h*_, *A*_*v*_ according to their vertical or horizontal orientation in our near-normal incidence experiment (see “Methods” section). Finally, solving the integrals in Eq. (3) results in the aforementioned frequency components of the correlation trace and the total signals

5 $${S}_{0\omega }^{{{{{{{{\rm{real}}}}}}}}}=	\; 4\left({A}_{h}^{4}\;{\cos }^{2}\;{\varphi }_{12}\;{\cos }^{2}\;{\varphi }_{23}+{A}_{v}^{4}\;{\sin }^{2}\;{\varphi }_{12}\;{\sin }^{2}\;{\varphi }_{23}\right)\\ 	+4{A}_{h}^{2}{A}_{v}^{2}\;{\sin }^{2}\left({\varphi }_{12}+{\varphi }_{23}\right)\\ {S}_{1\omega }^{{{{{{{{\rm{real}}}}}}}}}=	\; 8\cos \;\left(\omega {{\Delta }}t\right)\sin \left({\varphi }_{12}+{\varphi }_{23}\right)\\ 	\cdot \left({A}_{h}^{3}{A}_{v}\cos {\varphi }_{12}\cos {\varphi }_{23}+{A}_{h}{A}_{v}^{3}\sin {\varphi }_{12}\sin {\varphi }_{23}\right)\\ {S}_{2\omega }^{{{{{{{{\rm{real}}}}}}}}}=	\; 2\cos \;\left(2\omega {{\Delta }}t\right){A}_{h}^{2}{A}_{v}^{2}\sin \left(2{\varphi }_{12}\right)\sin \left(2{\varphi }_{23}\right)\\ {S}^{{{{{{{{\rm{real}}}}}}}}}=	\; \int\nolimits_{-\infty }^{+\infty }{S}_{0\omega }^{{{{{{{{\rm{real}}}}}}}}}+{S}_{1\omega }^{{{{{{{{\rm{real}}}}}}}}}+{S}_{2\omega }^{{{{{{{{\rm{real}}}}}}}}}\,{{{{{\rm{d}}}}}}t,$$

6 $${S}_{0\omega }^{{{{{{{{\rm{virt}}}}}}}}}=	\; 2\left({A}_{h}^{4}\;{\cos }^{2}\;{\varphi }_{12}\;{\cos }^{2}\;{\varphi }_{23}+{A}_{v}^{4}\;{\sin }^{2}\;{\varphi }_{12}\;{\sin }^{2}\;{\varphi }_{23}\right)\\ 	+2{A}_{h}^{2}{A}_{v}^{2}\sin \left(2{\varphi }_{12}\right)\sin \left(2{\varphi }_{23}\right)\\ {S}_{1\omega }^{{{{{{{{\rm{virt}}}}}}}}}=	\; 4\,\cos \;\left(\omega {{\Delta }}t\right)\;\sin \left({\varphi }_{12}+{\varphi }_{23}\right)\\ 	\cdot \left({A}_{h}^{3}{A}_{v}\cos {\varphi }_{12}\cos {\varphi }_{23}+{A}_{h}{A}_{v}^{3}\sin {\varphi }_{12}\sin {\varphi }_{23}\right)\\ {S}_{2\omega }^{{{{{{{{\rm{virt}}}}}}}}}=	\; 2\cos \left(2\omega {{\Delta }}t\right){A}_{h}^{2}{A}_{v}^{2}\\ 	\cdot \left({\cos }^{2}{\varphi }_{12}{\sin }^{2}{\varphi }_{23}+{\sin }^{2}{\varphi }_{12}{\cos }^{2}{\varphi }_{23}\right)\\ {S}^{{{{{{{{\rm{virt}}}}}}}}}=	 \int\nolimits_{-\infty }^{+\infty }{S}_{0\omega }^{{{{{{{{\rm{virt}}}}}}}}}+{S}_{1\omega }^{{{{{{{{\rm{virt}}}}}}}}}+{S}_{2\omega }^{{{{{{{{\rm{virt}}}}}}}}}\,{{{{{\rm{d}}}}}}t,$$

corresponding to real and virtual transitions, which now include the angular dependence on the orientation of the OTDs. Most importantly, they reveal non-vanishing contributions to the oscillating 1*ω* and 2*ω* components. We omitted the time-dependency of the electric field and its amplitude in the above equations for reasons of readability.

**Fig. 3:**
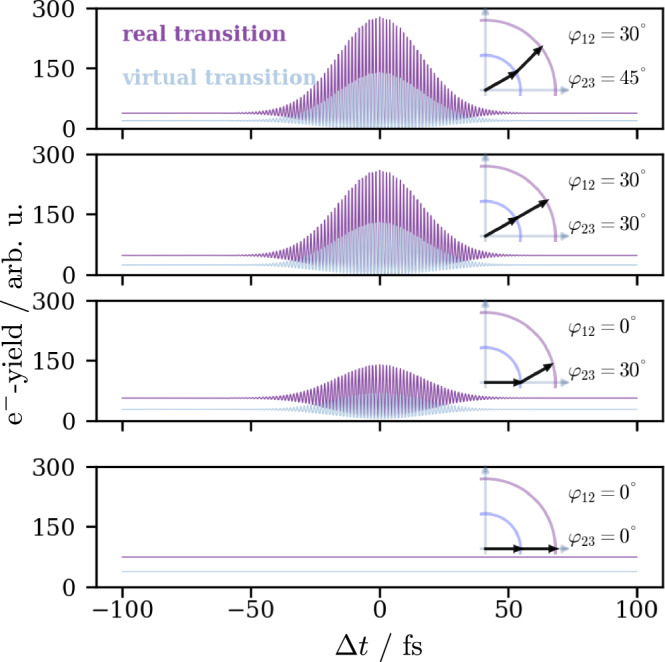
OTD-orientation dependent cross-polarized correlation traces. Simulated cross-correlation traces for different angles between the electric field and the optical transition dipoles for real (purple) and virtual (light blue) transitions. The different orientations alter the overall correlation signal strength. Supplementary Figure 4 contains simulations for additional angle combinations.

With this model, we are able to simulate time-dependent correlation traces for two cross-polarized pulses, which are exemplarily shown in Fig. 3 for the angle combinations {*φ*_12_, *φ*_23_} = {30^∘^, 45^∘^}, {30^∘^, 30^∘^}, {0^∘^, 30^∘^}, {0^∘^, 0^∘^}. Supplementary Figure 4 shows further simulations for additional angle combinations. The resulting curves show the coherent interference fringes, which were observed in the experiment, for both real and virtual intermediate states. The special case of one pulse aligning perfectly with both OTDs shown in the bottom of the figure results in a constant signal that is caused by two-photon excitation from the same laser pulse, in this example *E*_*h*_. The angular dependence causes differing intensities of the photoemission yield for both real and virtual transitions. Even when one of the pulses perfectly aligns with one of the OTDs (see {*φ*_12_, φ_23_} = {0^∘^, 30^∘^} in Fig. 3, bottom), a correlation trace with an interference pattern can be produced. This behavior leads to the question, if this model can be used to determine the angles *φ*_12_ and *φ*_23_ for real solids and therefore get information on the coupling between different states and the orientation of their orbitals as well as possible hybridization states of these orbitals. In the case of Ag(110), it is clear that the anisotropic orientation of the surface plays a role in light–matter interactions^27–29^ and we will discuss its influence on the OTDs below. At this point, the existence of OTDs with a fixed orientation explains the observed interference fringes in the correlation trace recorded on Ag(110) in the top of Fig. 1a. However, the concept of this fixed orientation should not apply to highly isotropic materials such as polycrystalline silver. In this case, the observed fringes shown in the bottom of Fig. 1a originate from a second mechanism (ii), which we address in the following.

### Phase-dependent effective light polarization

Our model presented above assumes a constant polarization of the two individual pulses for all time delays. In reality, however, the superposition of both pulses in space and time with a well-defined phase relation can be viewed as a single pulse with a continuously changing light polarization that depends on the phase difference between both individual pulses. During an optical cycle, the small time delay steps of 100 as control this phase *ϕ* between the two pulses *E* = *E*_*h*_(*ωt*) + *E*_*v*_(*ωt* + *ϕ*), resulting in a constantly changing effective polarization of the fields superimposed on the sample. In other words, the polarization alternates between linearly, elliptically, and circularly polarized light depending on *ϕ*. This naturally leads to a difference in photoemission yield for these polarizations due to the inherent polarization-dependent light absorption of matter and subsequently to an oscillation of the emission yield during temporal overlap of the pulses. This phenomenon is completely independent of the first mechanism (i) involving the OTDs.

We disentangle both mechanisms by considering a special case of our theoretical model for the transition dipole orientation.

Assuming an isotropic orientation of the OTDs, we simplify Eqs. (5) and (6) by an additional integration over all possible directions of *φ*_12_ and *φ*_23_

7 $${S}_{{{{{{{{\rm{iso}}}}}}}}}^{{{{{{{{\rm{real}}}}}}}}} =\int\nolimits_{0}^{2\pi }\int\nolimits_{0}^{2\pi }{S}^{{{{{{{{\rm{real}}}}}}}}}\,d{\varphi }_{12}d{\varphi }_{23},\\ {S}_{{{{{{{{\rm{iso}}}}}}}}}^{{{{{{{{\rm{virt}}}}}}}}} =\int\nolimits_{0}^{2\pi }\int\nolimits_{0}^{2\pi }{S}^{{{{{{{{\rm{virt}}}}}}}}}\,d{\varphi }_{12}d{\varphi }_{23}.$$

**Fig. 4:**
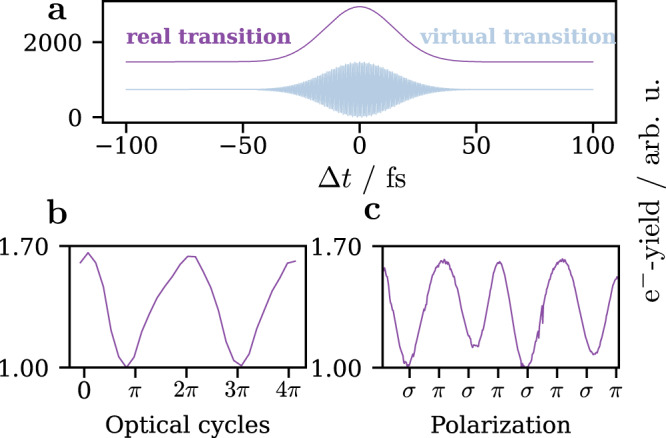
Phase-dependent effective light polarization on polycrystalline silver. **a** Simulated photoemission yield for an isotropic angle distribution. **b** Zoom into two optical cycles around time zero from Fig. 1b, normalized to the minimum signal. **c** Normalized photoemission yield for different polarizations impinging on polycrystalline silver.

The results are shown in Fig. 4a. Due to the averaging over the angular space, all coherent oscillations vanish and only the 0*ω* component remains for real intermediate states, while only the 2*ω* component contributes to the signal for virtual intermediate states (displayed in Fig. 4a). The latter however are mostly irrelevant for real polycrystalline metals as they typically do not exhibit any band gaps at least averaged over the illumination site. Therefore, we actually don’t expect any coherent oscillations, and only the ultrafast polarization switching mechanism remains in the signal.

The correlation trace recorded on polycrystalline silver shown at the bottom of Fig. 1a reproduces this special case due to the superposition of many crystal orientations within the illumination area of the rather big laser spots (*d*_beam_ ≈ 200 μm). To verify that only the phase-dependent polarization of the cross-polarized pulses is responsible for the oscillating signal, we take a closer look at the minima and maxima of the photoemission yield around time zero of the phase-stabilized correlation trace, which is shown in Fig. 4b. We compare it to a simple experiment with only one arm of the interferometer and a quarter wave-plate rotating from 0° to 360° shown in Fig. 4c. By normalizing the two graphs to their respective minima, it becomes evident, that the excitation conditions seem to be the same for both experiments, thus proving our assumption that the oscillating emission yield exclusively stems from the ultrafast polarization shift.

### Determining the OTD orientation by phase-averaging

Importantly, the phase-dependent light polarization does not only play a role in polycrystalline materials, but also in anisotropic materials such as Ag(110). In these cases, it is extremely challenging to disentangle both mechanisms (i) and (ii). This is, however, strictly necessary in order to extract reliable information on the orientation of the OTDs. Fortunately, the individual frequency components (0*ω*, 1*ω*, 2*ω*) exhibit distinctive dependence on the OTD orientation, and their concurrent contributions to the correlation trace are independent from each other. We make use of this by phase-averaging rather than phase-stabilizing the pulses during the experiment (see “Methods”), resulting in the exclusive signal of the non-oscillating 0*ω* component, while at the same time suppressing the ultrafast polarization switching. Phase-averaging additionally has the benefit of a greatly reduced complexity of the experimental setup as well as a substantial increase in statistics during the data acquisition, making the findings of this work more easily accessible in the conventional and widespread time-resolved but phase-averaged two-photon photoemission spectroscopy (tr-2PPE). Ultimately, we extract the OTD orientation from our model by simulating the *relative height* of the correlation trace solely comprised of the 0*ω* component that is typically recorded in conventional tr-2PPE. The relative height refers to the difference of the maximum signal for optimal pulse overlap at time zero Δ*t* → 0 and the constant background signal resulting from the two pulses driving the two-photon photoemission process individually. The latter constitutes the photoemission signal at time delays Δ*t* → *∞*, when the two pulses are completely separated. In the following, we illustrate this for momentum resolved data recorded on the surface of our Ag(110) crystal.

**Fig. 5:**
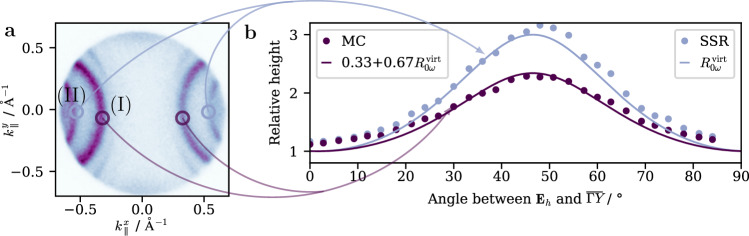
Momentum resolved relative height of the cross-polarized correlation trace on Ag(110). **a** Constant energy map at *E*_final_ = 6.2 eV showing the Mahan cone transition (I) and the surface scattering resonance (II)^30^, representing transitions via virtual intermediate states. **b** The relative height dependent on the angle *φ* between *E*_*h*_ and the $\overline{{{\Gamma }}}\overline{Y}$-direction for both features (dots), extracted and averaged from the circular regions of interest depicted in (**a**). The blue line shows the simulated relative height ${R}_{0\omega }^{{{{{{{{\rm{virt}}}}}}}}}$ (Eq. (8)). The purple line represents the relative height for a *sp*^2^-hybridized orbital, calculated by assigning an orbital nature to the different cases of our model.

Figure 5a displays the photoemission yield dependent on the parallel momentum of the photoelectrons with a final state energy of *E*_final_ = 6.2 eV. Here, we focus on the two outer parabolic features (I) and (II), which have been previously identified as a bulk interband transition (I) and a so-called *surface scattering resonance* (II)^30^. They have been previously reported for one-photon photoemission using a photon energy of 5.9 eV. Therefore, we consider here the case of virtual intermediate states with an identical orientation of the transition dipoles *φ*_12_ = *φ*_23_ = *φ* for our discussion. Using Eq. (6) we can ultimately calculate the relative height

8 $${R}_{0\omega }^{{{{{{{{\rm{virt}}}}}}}}}=\frac{{S}_{0\omega }^{{{{{{{{\rm{virt}}}}}}}}}({{\Delta }}t\to 0)}{{S}_{0\omega }^{{{{{{{{\rm{virt}}}}}}}}}({{\Delta }}t\to \infty )}=1+\frac{{\sin }^{2}\left(2\varphi \right)}{{\sin }^{4}\left(\varphi \right)+{\cos }^{4}\left(\varphi \right)}.$$

Figure 5b displays the relative height depending on the angle *φ* between the vertically oriented pulse and the $\overline{{{\Gamma }}}\overline{Y}$-direction of the surface, which is controlled by a rotating half-wave plate. The relative height was extracted from the momentum resolved data by dividing the image recorded at time zero by the one taken for a delay far from time zero and subsequently defining suitable regions of interests to separate the signals of (I) (blue dots) and (II) (purple dots). The blue line is the relative height ${R}_{0\omega }^{{{{{{{{\rm{virt}}}}}}}}}$ calculated above and it reproduces the extracted data for feature (II) astonishingly well. This suggests that the transition dipole for the surface scattering resonance aligns with the $\overline{{{\Gamma }}}\overline{Y}$-direction of the surface. This resonance originates from a Bloch state, where the final state and initial state have the same momentum modulo a reciprocal lattice vector ${\overrightarrow{k}}_{f}={\overrightarrow{k}}_{i}+\overrightarrow{G}$. This state scatters at the surface resulting in additional surface-derived reciprocal lattice vectors ${\overrightarrow{k}}_{f}={\overrightarrow{k}}_{i}+{\overrightarrow{G}}_{\parallel }$, which differ from their bulk counterparts due to a lattice mismatch between the surface projected bulk Brillouin zone and the surface Brillouin zone. In this case, with the lattice constant *a*, the surface reciprocal lattice vector ${\overrightarrow{G}}_{\parallel }=\left(0,0,\pm \frac{2\pi }{a}\right)$ leading to the surface scattering resonance (II), aligns as well with the $\overline{{{\Gamma }}}\overline{Y}$-direction^30^. This leads us to conclude that the transition dipole for an excitation with 3.1 eV is closely tied to the surface reciprocal lattice vector.

Similarly, we can assume that the transition dipole of the interband transition relates to its bulk reciprocal lattice vector ${\overrightarrow{G}}_{111}$, or rather its projection onto the surface. Coincidently, the projection is parallel to the $\overline{{{\Gamma }}}\overline{Y}$-direction as well. However, the relative height for (I) (purple dots in Fig. 5b) is clearly lower than the one of the surface scattering resonance. To interpret this result, we associate the directivity of the transition dipoles with an orbital character. In particular, when we can assign a transition a fixed orientation of the transition dipole, we treat it as a *p*-type orbital. The mathematical description of the polycrystalline silver case, averaged over the illumination area of the laser spots, in the previous section on the other hand suggests an interpretation in terms of an *s*-type orbital due to the isotropic distribution of transition dipoles. For this, the relative height delivers 1 for all angles, since the 0*ω* component does not contribute more to the signal than the constant background (see Fig. 4a).

By combining the two cases of fixed (*p*-orbitals) and isotropic (*s*-orbitals) OTD orientation, we can now emulate hybridization between *s*- and *p*-type orbitals and interpret the relative height as a superposition of both OTDs of the bulk bands according to their degree of *sp*-hybridization. For this, let us consider the three typical cases of *sp*, *sp*^2^, and *sp*^3^ hybridization, which exhibit distinct fractional characters of *s*- and *p*-type orbitals. Specifically, we consider the case of *sp*^2^-hybridization, where the orbital character is a mixture of 33% parts *s*-type and 67% *p*-type. We can calculate the relative height of transition (I) with a superposition of the relative heights for fixed and isotropically oriented OTDs modulated by the fractional orbital character for *sp*^2^-hybridization. This results in the total relative height of ${R}_{0\omega }^{(I)}=0.33\cdot 1+0.67\cdot {R}_{0\omega }^{{{{{{{{\rm{virt}}}}}}}}}$ for a third of the *sp*^2^-orbital comprised of the *s*-orbital and the remaining part stemming from two *p*-orbitals. We display this relative height as a purple line in Fig. 5b, which fits remarkably well to the measured relative height of (I). Hence, we surmise that the transition dipole of the interband transition is governed by the orbital nature of *sp*^2^-hybridized bulk bands with an alignment along the $\overline{{{\Gamma }}}\overline{Y}$-direction of the surface.

In conclusion, we uncovered the existence of coherent interference effects in the optical response of a poly-and a (110)-oriented single crystalline silver surface after optical excitation with two phase-stabilized fs-light pulses with orthogonal linear polarization. Our findings are discussed in the framework of the density matrix formalism and can be attributed to different aspects of the light–matter interaction. On the one hand, the constantly changing phase between the cross-polarized light pulses during the cross-correlation experiments leads to a constantly changing light-absorption of both silver samples at each step of the cross-correlation trace, which causes an oscillating signal in the two-pulse correlation trace. The most important mechanism, however, is the intrinsic coupling of the non-interfering light fields within the single crystalline material. This coupling can only be explained by the existence of OTDs with fixed orientations with respect to the silver lattice.

Our work has clearly demonstrated the important role of the orientation of the OTDs for the electronic response of free electron-like metals. This does not only advance our understanding of the light–matter interaction in metals. It also allows us to quantify the orbital character of valence states by the orientations of their OTDs, as demonstrated for the metallic valence states of the Ag(110) surface. This approach can potentially open new avenues towards the experimental characterizations of the orbital character of valence and conduction states of complex metallic or semi-metallic quantum materials such as Weyl semimetals or other types of topological materials.

## Methods

### Sample preparation

The Ag(110) single crystal was prepared by Ar^+^-sputtering and subsequent annealing. The Argon gas (pressure ≈ 6 × 10^−6^ mbar) was ionized with a current of 10 mA and accelerated towards the sample with a voltage of 1500 V. The surface normal of the sample was aligned with the optical axis of the sputter gun for normal incidence sputtering. The sample was subsequently heated to 550 ^∘^C. The duration of a sputtering cycle was typically in the range of 20–30 min and the following annealing cycle twice the duration of the sputtering. The polycrystalline sample is a 100 nm thick target-sputtered silver film on an oxidized silicon wafer substrate.

### Experimental technique

Our momentum microscope is a photoemission electron microscope (Focus GmbH) combined with a time-of-flight delay line detector (Surface Concept GmbH) situated in an ultra-high vacuum chamber (*p* < 1 × 10^−9^ mbar). We work with an energy resolution of about Δ*E* ≳ 100 meV and a momentum resolution of Δ*k* ≳ 2 × 10^−2^ Å^−1^. With an additional mirror inside the electronic lens system we illuminate the sample under near-normal incidence (4^∘^ with respect to the surface normal), resulting in the in-plane electric field orientation.

We use the second harmonic of the output of a commercial Titanium-Sapphire Oscillator (Spectra Physics) operating at a central wavelength of 800 nm, resulting in pulses with a photon energy of 3.1 eV and a pulse duration of approximately 35 fs. The laser system emits pulses at a frequency of 75 MHz with a fluence of less than 1 μJ/cm^2^.

We employ an actively phase-stabilized cross-polarized Mach–Zehnder interferometer in order to record the correlation traces with time delay steps of 100 as between the two pulses. The polarization of one interferometer arm is rotated by 90^∘^ using a half-wave plate and the power of the second arm is reduced with a rotating gray filter to make up for the difference in reflectivities of the mirrors caused by the different polarization. Additionally, we use a second non-stabilized interferometer in order to perform the phase-averaged experiments. Here, a vibrating motor is attached to the mount of one of the mirrors in one interferometer arm. The high-frequency vibration displaces the mirror on the *μ*m-scale during the integration time of the experiment resulting in an effective averaging over tiny shifts in the delay between the two pulses.

## Supplementary information

Supplementary Information

## Data Availability

The data that support the findings of this study are available from the corresponding author upon request.

## Code availability

The code relevant for the evaluation of the experimental data and for simulating the correlation traces is available from the corresponding author upon request.

## References

[1] Brixner T (2005). Two-dimensional spectroscopy of electronic couplings in photosynthesis. Nature.

[2] Engel GS (2007). Evidence for wavelike energy transfer through quantum coherence in photosynthetic systems. Nature.

[3] Lee H, Cheng Y-C, Fleming GR (2007). Coherence dynamics in photosynthesis: Protein protection of excitonic coherence. Science.

[4] Collini E, Scholes GD (2009). Coherent intrachain energy migration in a conjugated polymer at room temperature. Science.

[5] Collini E (2010). Coherently wired light-harvesting in photosynthetic marine algae at ambient temperature. Nature.

[6] Panitchayangkoon G (2010). Long-lived quantum coherence in photosynthetic complexes at physiological temperature. Proc. Natl Acad. Sci. USA.

[7] Andrea Rozzi C (2013). Quantum coherence controls the charge separation in a prototypical artificial light-harvesting system. Nat. Commun..

[8] Falke SM (2014). Coherent ultrafast charge transfer in an organic photovoltaic blend. Science.

[9] Hong X (2014). Ultrafast charge transfer in atomically thin MoS_2_/WS_2_ heterostructures. Nat. Nanotechnol..

[10] Ceballos F, Bellus MZ, Chiu H-Y, Zhao H (2014). Ultrafast charge separation and indirect exciton formation in a MoS_2_–MoSe_2_ van der Waals heterostructure. ACS Nano.

[11] Rivera P (2015). Observation of long-lived interlayer excitons in monolayer MoSe_2_–WSe_2_ heterostructures. Nat. Commun..

[12] Long R, Prezhdo OV (2016). Quantum coherence facilitates efficient charge separation at a MoS_2_/MoSe_2_ van der Waals junction. Nano Lett..

[13] Stadtmüller B (2019). Strong modification of the transport level alignment in organic materials after optical excitation. Nat. Commun..

[14] Yong H (2018). Determining orientations of optical transition dipole moments using ultrafast X-ray scattering. J. Phys. Chem. Lett..

[15] Nordén B (1978). Applications of linear dichroism spectroscopy. Appl. Spectrosc. Rev..

[16] Lohmüller T, Erdmann M, Rubner O, Engel V (2003). Determination of transition dipole moments from time-resolved photoelectron spectroscopy. Eur. Phys. J. D. - At., Mol., Optical Plasma Phys..

[17] Grechko M, Zanni MT (2012). Quantification of transition dipole strengths using 1d and 2d spectroscopy for the identification of molecular structures via exciton delocalization: Application to *α*-helices. J. Chem. Phys..

[18] Mu T, Chen S, Zhang Y, Guo P, Chen H (2015). Determining the orientation of transition moments and depolarization by fluorescence polarizing angle spectrum. Opt. Express.

[19] Sturm K (1978). Band structure effects on the plasmon dispersion in simple metals. Eur. Phys. J. B.

[20] Hertel T, Knoesel E, Wolf M, Ertl G (1996). Ultrafast electron dynamics at Cu(111): Response of an electron gas to optical excitation. Phys. Rev. Lett..

[21] Ogawa S, Nagano H, Petek H, Heberle AP (1997). Optical dephasing in Cu(111) measured by interferometric two-photon time-resolved photoemission. Phys. Rev. Lett..

[22] Höfer U (1997). Time-resolved coherent photoelectron spectroscopy of quantized electronic states on metal surfaces. Science.

[23] Bauer M, Aeschlimann M (2002). Dynamics of excited electrons in metals, thin films, and nanostructures. J. Electron Spectrosc. Relat. Phenom..

[24] Merschdorf, M. *Femtodynamics in Nanoparticles: The Short Lives of Excited Electrons in Silver.* Dissertation, Universität Würzburg, Würzburg (2002).

[25] Spektor G (2019). Mixing the light spin with plasmon orbit by nonlinear light–matter interaction in gold. Phys. Rev. X.

[26] Merschdorf M, Kennerknecht C, Pfeiffer W (2004). Collective and single-particle dynamics in time-resolved two-photon photoemission. Phys. Rev. B.

[27] Borensztein Y, Mochan WL, Tarriba J, Barrera RG, Tadjeddine A (1993). Large anisotropy in the optical reflectance of ag(110) single crystals: Experiment and theory. Phys. Rev. Lett..

[28] Calmels L, Inglesfield JE, Arola E, Crampin S, Rasing T (2001). Local-field effects on the near-surface and near-interface screened electric field in noble metals. Phys. Rev. B.

[29] Li A (2021). Plasmonic photoemission from single-crystalline silver. ACS Photonics.

[30] Eul T, Braun J, Stadtmüller B, Ebert H, Aeschlimann M (2021). Spectroscopic evidence for a new type of surface resonance at noble-metal surfaces. Phys. Rev. Lett..

