# Supplementary material for: Coherent response of the electronic system driven by non-interfering laser pulses
Source: Nat Commun. 2022 Jun 9;13:3324. doi: 10.1038/s41467-022-30768-9 (PMC9184506; doi:10.1038/s41467-022-30768-9)
Supplement: Supplementary file 1 — Supplementary Information [file 41467_2022_30768_MOESM1_ESM.pdf]

Supplementary Information: Coherent response of the electronic system driven by non-interfering laser pulses

T. Eul et al.

Supplementary Note 1. TWO-PULSE CORRELATIONS WITH RANDOM PHASES

The recording of phase-stabilized correlation traces requires the experimental conditions, e.g. the pointing as well as the power of the excitation source, to remain stable over a period of several tens of hours for a successful experiment. Fortunately, it is possible to determine whether the optical transitions in a sample will exhibit anisotropic orientations of the optical transition dipoles (OTDs) in a less time-consuming manner. Such trial runs forego the active phase-stabilization and require only small exposure times per time delay step resulting in the interference of the two laser pulses with random phases. The corresponding correlation traces with two cross-polarized pulses, therefore, exhibit random interference maxima or minima during temporal overlap, which only manifest for samples with anisotropic orientations of the OTDs. On a side note, experiments with two collinearly polarized pulses without active phase-stabilization usually contain an additional device, which slightly oscillates the position of a single mirror in one arm of the interferometer to average over these randomly generated phases between the two pulses. Here, we intentionally do not destroy the random phase in order to efficiently probe the existence of an anisotropic orientation of the OTDs in other materials than Ag(110). The following sections display these additional trial runs on single crystalline Ag(110), Au(110) and Cu(110) samples integrated over all energies and the total available momentum space recorded with our time-of-flight momentum microscope. All three cases exhibit an anisotropic orientation of the OTDs of the transitions addressed with our photon energy of 3.1 eV. The strongest effect is visible for the gold sample, whereas the copper sample only has minor oscillations in the correlation trace.

1.1. Ag(110)

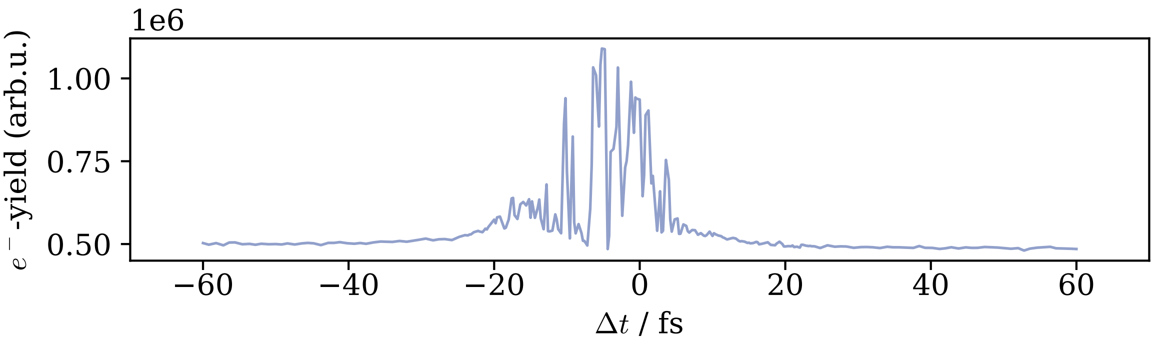

Supplementary Fig. 1. Phase-averaged correlation trace with two cross-polarized pulses recorded on Ag(110) for an exposure time of 2 s per time delay step with a step size of 1 fs. The photon energy is 3.1 eV and the traces are integrated over all energies and the whole accessible momentum space.

1.2. Au(110)

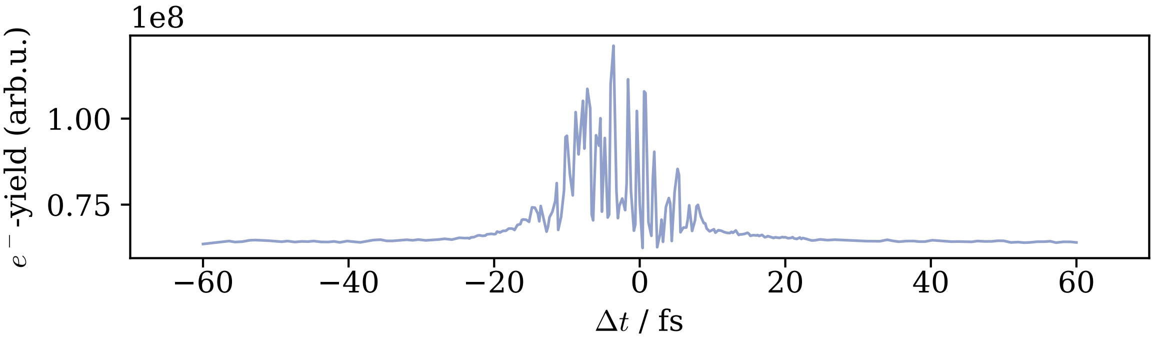

Supplementary Fig. 2. Phase-averaged correlation trace with two cross-polarized pulses recorded on Au(110) for an exposure time of 5 s per time delay step with a step size of 1 fs. The photon energy is 3.1 eV and the traces are integrated over all energies and the whole accessible momentum space.

1.3. Cu(110)

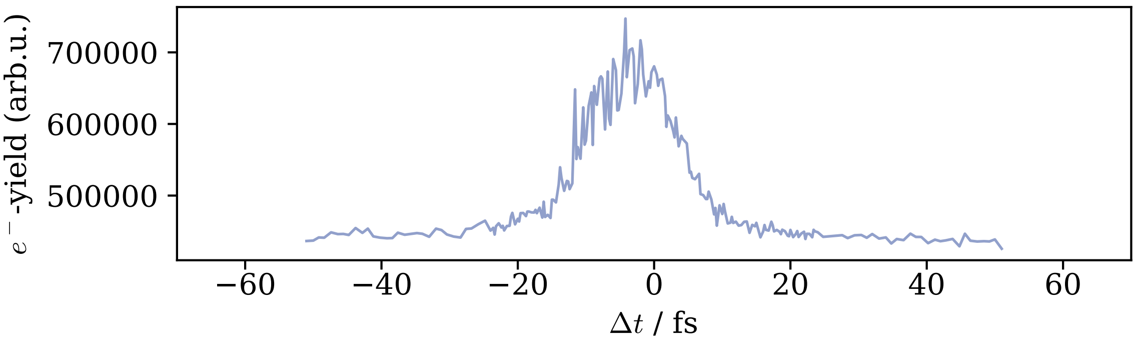

Supplementary Fig. 3. Phase-averaged correlation trace with two cross-polarized pulses recorded on Cu(110) for an exposure time of 1 s per time delay step with a step size of 1 fs. The photon energy is 3.1 eV and the traces are integrated over all energies and the whole accessible momentum space.

Supplementary Note 2. ADDITIONAL SIMULATIONS

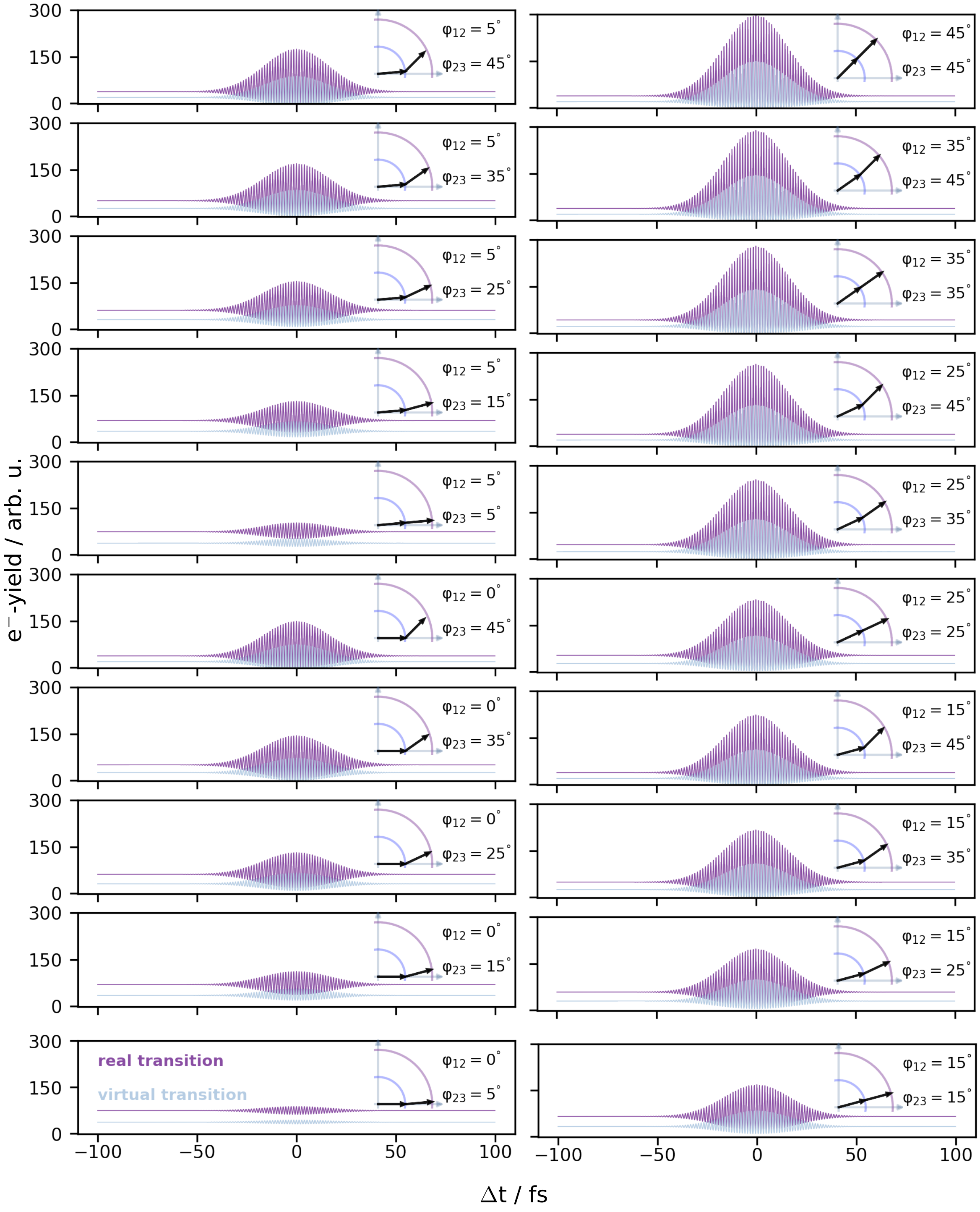

Supplementary Fig. 4. Additional simulations of cross-polarized correlation traces for different orientations of the optical transition dipoles expressed by the angles φ_{12} and φ_{23} according to their definition in Fig. 2c) of the main manuscript. The correlation signals for transitions via real and virtual intermediate states are displayed in purple and light blue respectively. The different orientations of the optical transition dipoles impact the relative height of the correlation signal with its maximum for $\varphi_{12} = \varphi_{23} = 45$, which emulates the case of two pulses with parallel polarization.
